# Role of mTOR Signaling Cascade in Epidermal Morphogenesis and Skin Barrier Formation

**DOI:** 10.3390/biology11060931

**Published:** 2022-06-19

**Authors:** Juan Wang, Sabine A. Eming, Xiaolei Ding

**Affiliations:** 1 Institute of Geriatrics (Shanghai University), Affiliated Nantong Hospital of Shanghai University (The Sixth People’s Hospital of Nantong), School of Medicine, Shanghai University, Nantong 226011, China; juanw@shu.edu.cn; 2 Shanghai Engineering Research Center of Organ Repair, School of Medicine, Shanghai University, Shanghai 200444, China; 3 Department of Dermatology, University of Cologne, 50937 Cologne, Germany; 4 Cologne Excellence Cluster on Cellular Stress Responses in Aging-Associated Diseases (CECAD), University of Cologne, 50674 Cologne, Germany; 5 Center for Molecular Medicine Cologne (CMMC), University of Cologne, 50931 Cologne, Germany; 6 Institute of Zoology, Developmental Biology Unit, University of Cologne, 50674 Cologne, Germany

**Keywords:** epidermis, epidermal morphogenesis, skin barrier, mTOR, mouse models

## Abstract

**Simple Summary:**

The skin epidermis is a stratified multilayered epithelium that provides a life-sustaining protective and defensive barrier for our body. The barrier machinery is established and maintained through a tightly regulated keratinocyte differentiation program. Under normal conditions, the basal layer keratinocytes undergo active proliferation and migration upward, differentiating into the suprabasal layer cells. Perturbation of the epidermal differentiation program often results in skin barrier defects and inflammatory skin disorders. The protein kinase mechanistic target of rapamycin (mTOR) is the central hub of cell growth, metabolism and nutrient signaling. Over the past several years, we and others using transgenic mouse models have unraveled that mTOR signaling is critical for epidermal differentiation and barrier formation. On the other hand, there is increasing evidence that disturbed activation of mTOR signaling is significantly implicated in the development of various skin diseases. In this review, we focus on the formation of skin barrier and discuss the current understanding on how mTOR signaling networks, including upstream inputs, kinases and downstream effectors, regulate epidermal differentiation and skin barrier formation. We hope this review will help us better understand the metabolic signaling in the epidermis, which may open new vistas for epidermal barrier defect-associated disease therapy.

**Abstract:**

The skin epidermis, with its capacity for lifelong self-renewal and rapid repairing response upon injury, must maintain an active status in metabolism. Mechanistic target of rapamycin (mTOR) signaling is a central controller of cellular growth and metabolism that coordinates diverse physiological and pathological processes in a variety of tissues and organs. Recent evidence with genetic mouse models highlights an essential role of the mTOR signaling network in epidermal morphogenesis and barrier formation. In this review, we focus on the recent advances in understanding how mTOR signaling networks, including upstream inputs, kinases and downstream effectors, regulate epidermal morphogenesis and skin barrier formation. Understanding the details of the metabolic signaling will be critical for the development of novel pharmacological approaches to promote skin barrier regeneration and to treat epidermal barrier defect-associated diseases.

## 1. Introduction

The skin is composed of three layers: the epidermis, dermis and the deeper subcutaneous tissue. The epidermis is a stratified squamous epithelium that serves as the primary interface between the body and the outside environment, providing a life-sustaining permeability barrier by preventing water and heat loss and protecting the body from various environmental insults. In response to danger signals or injury, the epidermis needs continuous self-renewal throughout life to maintain the barrier function [1]. Disruptions in the epidermal barrier integrity frequently precede and accompany the development of various skin diseases [2].

Studies with gene-targeted mutations in mice have unraveled important regulatory networks over the past decades, including signaling pathways and transcriptional factors that control epidermal morphogenesis and the barrier formation [3]. Among these, metabolic modulations have been recently demonstrated as crucial regulators of epidermal development. Particularly, the mechanistic target of rapamycin (mTOR) complexes and their upstream and downstream mediators constitute the major signaling cascade that regulates cell growth and metabolism [4]. Furthermore, their aberrant regulations have been implicated in the development of multiple skin pathological disorders, including impaired wound healing [5], atopic dermatitis (AD) [6] and the other hyperproliferative skin disorders [6,7,8]. In this review, we describe formation of epidermal barrier and delineate the transduction of mTOR signaling, with a particular focus on the involvement of mTOR complexes and other components of mTOR signaling network in epidermal morphogenesis and skin barrier function gained from transgenic mouse models. A comprehensive understanding of the molecular mechanisms underlying mTOR signaling pathway in epidermal barrier can aid in the development of novel targeted therapies for epithelial barrier defects. Of note, the epidermal structure and function are distinct between mice and humans in many aspects [9]. As a result, it is important to consider that the observed phenotypes with mouse studies might have different functional mechanisms under human skin physiological and pathological conditions.

## 2. Epidermal Barrier Formation

The epidermis is developed from a single-layered epithelium, originated from the surface of the ectoderm at around embryonic day 9.5 (E9.5) in mice. Signals from dermal mesenchymal cells together with intracellular master regulators, such as p63, direct the epithelial cells towards differentiation and stratification to yield the spinous, granular and cornified layers. A fully functional epidermis is nearly completed by E17.5 [10].

**Figure 1:**
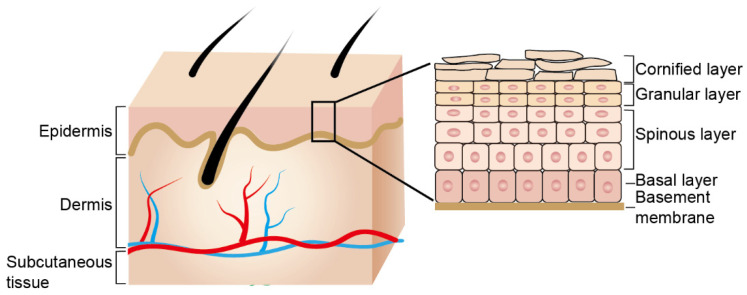
Structure of the skin and the skin epidermis. Skin is composed of three layers: epidermis, dermis and the deeper subcutaneous tissue. The dermis and epidermis are separated by basement membrane, where basal layer keratinocytes adhere. Keratinocytes of the basal layer undergo a terminal differentiation process and organize into distinct layers: spinous, granular and cornified layers.

In postnatal life, the epidermis continuously rejuvenates itself to replenish the shed corneocytes, dying or damaged cells upon injury. To achieve this, the proliferating keratinocyte stem/progenitor cells in the basal layer lose their mitotic activity, migrate upwards and differentiate into spinous layers. After detaching from the basement membrane (BM), the cells start the terminal differentiation program, known as cornification or keratinization. Dynamic cell morphological changes and sequential expression of specific markers, including keratins and barrier proteins, are characterized by the terminal differentiation program. Ultimately, the flattened squames form the outmost cornified layer, which provides the major protective barrier function of the skin [11] (Figure 1). The homeostasis of epidermal barrier integrity is a complex and precisely coordinated process. Disturbances of this process can lead to skin barrier impairment, which is considered the hallmark of the diverse inflammation skin diseases, such as AD and psoriasis [2,11].

## 3. Overview of the mTOR Signaling Network: Key Players and Mechanisms

The mTOR protein is atypical serine/threonine kinase and was firstly identified through genetic screening as a nutrient sensor, regulating cell growth and proliferation [12,13]. Subsequent investigations have largely expanded our knowledge about mTOR, including that it is an evolutionarily conserved protein and coordinates diverse functions in eukaryotes [14,15].

**Figure 2:**
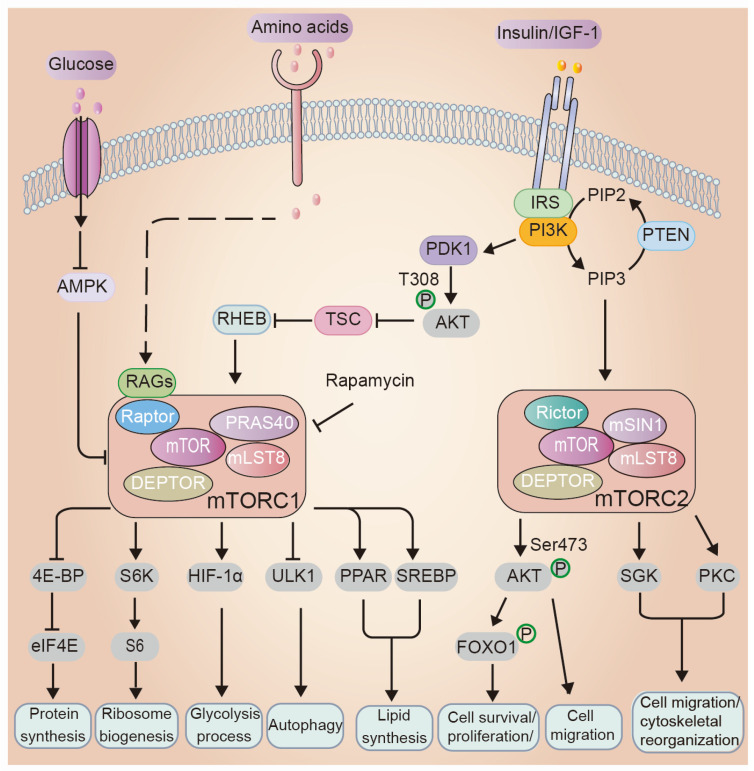
Overview of mTOR signaling network. Amino acids, growth factors, oxygen and the cellular energy are the major inputs inducing mTORC1 activity. Upon stimulating by the growth factors such as insulin/insulin-like growth factor 1 (IGF-1), the receptor tyrosine kinase phosphorylates PI3K and subsequently activates PI3K-PDK1-AKT signaling cascades. The activated AKT phosphorylates tuberous sclerosis complex 2 (TSC2) and inhibits TSC1 and TSC2 complex formation. This further inactivates TSC1/2 and releases Ras homolog enriched in brain (RHEB), which in turn binds and activates mTORC1. The activated mTORC1 phosphorylates downstream effectors, such as S6K and 4E-BP1, governing several crucial cellular processes including protein synthesis, lipid metabolism, ribosome biosynthesis and autophagy. Additionally, in response to amino acids, RAS-related GTP binding proteins (RAGs) form obligate heterodimers, which then bind RAPTOR to stimulate mTORC1 activity. The upstream regulators of mTORC2 activation are less well defined but supposed through PI3K. Activated mTORC2 phosphorylates the AGC kinase family members, mainly protein kinase C (PKC), serum glucocorticoid-regulated kinase (SGK) and AKT, which primarily regulate cell metabolic processes, survival, migration and cytoskeletal reorganization.

By interacting with various regulatory proteins, mTOR forms two complexes with distinct structure and function, referred to as mTOR complex 1 (mTORC1) and mTORC2 (Figure 2). mTORC1 is sensitive to rapamycin and consists primarily of mTOR, DEP-domain-containing mTOR-interacting protein (DEPTOR), mammalian lethal with SEC13 protein 8 (mLST8), proline-rich AKT substrate 40 kDa (PRAS40), and its unique scaffold regulatory-associated protein of mTOR (RAPTOR) [14,15]. Like mTORC1, the core components of mTORC2 include mTOR, DEPTOR and mLST8. Meanwhile, mTORC2 is distinguished by the presence of a rapamycin-insensitive companion of mTOR (RICTOR), MAPK-interacting protein 1 (mSIN1) and resistance to acute rapamycin treatment [14,15].

### 3.1. Regulations of mTOR Signaling

mTORC1 regulates cell growth and metabolism by responding to multiple upstream inputs such as growth factors, nutrients, energy and oxygen stress [15,16] (Figure 2). In response to growth factors, such as insulin/insulin-like growth factor 1 (IGF-1), PI3K phosphorylates phosphatidylinositol-4, 5-bisphosphate (PIP2), leading to the production of phosphatidylinositol-3,4,5-triphosphate (PIP3). Accumulation of PIP3 therewith recruits several kinases, including phosphoinositide-dependent kinase (PDK1). Subsequently, PDK1 phosphorylates AKT at threonine 308 (T308). The activated AKT further phosphorylates and inhibits tuberous sclerosis complex (TSC) heterodimer TSC1-TSC2, releasing the inhibition of Ras homolog enriched in brain (RHEB), which binds and activates mTORC1 [17,18,19]. Phosphatase and tensin homolog located on chromosome 10 (PTEN) is another important upstream negative mediator of mTORC1, as it can reverse PI3K functions resulting in decreased effective concentration of PIP3 [15]. Additionally, mTORC1 activity can also be induced by nutrients, especially amino acids [20]. In amino acid-adequate conditions, RAS-related GTP binding proteins (RAGs) form obligate heterodimers, which then bind RAPTOR to stimulate mTORC1 activity [21]. Furthermore, energy is another regulator of the status of mTORC1, e.g., glucose removal and oxidative stress can activate TSC signaling and dampen mTORC1 activity [16].

In contrast to mTORC1, the specific molecular mechanism by which mTORC2 is regulated is less understood. However, it is known that in response to growth factor stimuli, mTORC2 is primarily controlled by the PI3K pathway. PIP3 can directly bind the unique mTORC2 component mSIN1 to alleviate mSIN1-modulated auto-inhibition [22,23]. Insulin-induced PI3K can also activate mTORC2 by promoting the mTORC2–ribosome interaction, which is necessary for mTORC2 activation [24,25]. Furthermore, it is also important to note the cross-talk between mTORC1 and mTORC2. mTORC2 phosphorylates AKT protein on Ser473, which is required for full activation of AKT [26]. Hence, mTORC2 may positively regulate mTORC1 through AKT. As mTORC1 downregulates insulin-PI3K-AKT via IRS degradation, mTORC2 is likewise controlled by mTORC1 via a negative feedback loop [26]. Meanwhile, mTORC1 stabilizes GRB10, an insulin inhibitory factor, resulting in feedback regulation of mTORC2 [27].

### 3.2. Cellular Processes Regulated by mTOR

Upon activation, mTORC1 enhances primarily anabolic metabolism, including protein, nucleotide and lipid biosynthesis and glucose metabolism and decreases catabolic metabolism including suppression autophagy by phosphorylating several related effectors (Figure 2). Ribosomal protein S6 kinase (S6K) and eukaryotic initiation factor 4E (eIF-4E) binding protein (4E-BP) represent the best-characterized downstream effectors of mTORC1. Under conditions of favorable nutrient availability, mTORC1 phosphorylates 4E-BP, which consequently dissociates from eIF4E, a positive regulator of 5′-cap-dependent translation, leading to increased translation initiation [28,29,30]. Similarly, mTORC1 can phosphorylate S6K on T389, which further activates ribosomal protein S6 (S6), leading to enhanced ribosome biogenesis and translation of mRNA [16]. Activated mTORC1 enhances sterol regulatory element binding protein 1/2 (SREBP1/2) and peroxisome proliferator-activated receptor-γ (PPAR-γ)-mediated transcriptional activity, thereby driving lipid synthesis [31,32,33]. Further, mTORC1 activity can also drive de novo synthesis of nucleotides, including purine synthesis by activating transcription factor ATF4-mediated MTHFD2 expression, and pyrimidine synthesis by activating carbamoyl-phosphate synthetase 2, aspartate transcarbamoylase, dihydroorotase (CAD) in a S6K1-dependent manner [34,35]. In addition to its roles in biosynthesis, mTORC1 is able to enhance the expression of hypoxia inducible factor 1α (HIF-1α), which is a transcriptional factor and promotes the glycolysis process by regulating expression of glycolytic enzymes [36,37]. In nutrient replete conditions, mTORC1 phosphorylates ULK1 on Ser757 and ATG13 on Ser259, two key kinases that initiate autophagy, consequently preventing autophagy [38,39,40,41].

While the cellular processes controlled by mTORC2 signaling are less understood than mTORC1, accumulating evidence shows that mTORC2 contributes to modulating cell migration, metabolic processes, cell survival and proliferation [16]. When mTORC2 is activated, it phosphorylates the hydrophobic motifs of AKT, serum/glucocorticoid-regulated kinase 1 (SGK1), and protein kinase C (PKC), which are members of AGC family kinases (Figure 2). Phosphorylation of Ser473 in the hydrophobic motif of AKT is the best characterized effector of mTORC2 [42]. The phosphorylated and activated AKT further phosphorylates its downstream effectors, including FoxO1, which are crucial for cell survival and proliferation [16].

## 4. mTOR Signaling in Epidermal Morphogenesis and Barrier Formation

Although the general role and function of mTOR signaling at the cellular level is relatively well characterized, the physiological function of mTOR signaling in specific tissues and organs is still not well defined. Given the fundamental role of mTOR signaling in cell growth and metabolism, constitutive knockout of the core component often results in embryonic lethality [43,44]. Conditional knockout mouse models based on the Cre/loxP technique have been demonstrated as a powerful tool in studying gene function in vivo [45]. During the last two decades, studies in transgenic and knockout mouse models have greatly contributed to our understanding of the physiological and pathological functions of mTOR signaling cascade [46]. Recent studies with gene-targeted mutation in mice have highlighted the important role of mTOR signaling in regulating epidermal morphogenesis and skin barrier formation. In this part, we will discuss recent findings in the regulation of epidermal barrier by mTOR signaling inspired by mouse model studies. For skin phenotypes of mice with altered mTOR signaling activity, please refer to Table 1.

### 4.1. The Role of mTOR Upstream Regulators in Epidermal Morphogenesis and Barrier Formation

IGF-1 and insulin represent the major growth factors that can promote mTOR activity and regulate tissue growth and development. They mediate their effects by binding to IGF receptors (IGF-1R, IGF-2R) or insulin receptor (IR), respectively. Studies with their deficient mice showed that IR is not required for epidermal development, whereas mice deficient for IGF-1 or IGF-1R display severe epidermal hypoplasia [47,88]. In line with body-wide IR deletion, epidermal-specific IR mutant mice display no obvious skin barrier defects macroscopically, even though the histological structure of epidermis is hypoplastic. By contrast, epidermal-specific IGF-1R knockout mice exhibit fragile and translucent skin morphology and compromised skin barrier [49,50], suggesting a critical role for IGF-1 signaling in regulating epidermal morphogenesis. Intriguingly, mice with double mutants of IR/IGF-1R in epidermis show a much more severe phenotype than IGF-1R deletion mice and are not able to survive due to gross epidermis defects, suggesting that insulin and IGF-1 have cooperative roles in contributing to epidermal morphogenesis [50].

DiGiovanni J et al. generated a mouse model with enforced expression of IGF-1 in epidermis. In contrast to IGF-1 deletion, these mice display a wrinkled and thickened epidermis. In addition, persistent overexpression of IGF-1 leads to epidermal hyperplasia, hyperkeratosis and cutaneous tumorigenesis, identifying the high concentration of IGF-1 resulting in the malignant behaviour of skin papillomas [48]. With CRISPR/Cas9 genome editing technology and a three-dimensional organoid model, Muraguchi et al. showed that IGF-1R deficiency in human keratinocytes disrupts epidermal homeostasis and stem cell maintenance in organic culture [51], demonstrating a conserved role of IGF-1 signaling in human epidermis. These findings indicate that insulin/IGF-1 signaling is essential for epidermal morphogenesis and skin barrier formation. In addition, IGF-1 may control epidermal barrier function in a cell-autonomous manner. In ulcerative skin lesions of patients, the basal layer lacks IGF-1 expression compared to health skin [89]. Interestingly, a recent study showed that sustained release of IGF-1 leads to the enhanced wound re-epithelialization in diabetic mice [90].

Glucose is the primary source of cellular energy and glucose deprivation can suppress mTORC1 signaling through the major energy sensor AMP-activated protein kinase (AMPK) [16]. Glucose transporter 1(GLUT1) is the main glucose transport expressed in keratinocytes [52]. Although GLUT1 deletion impairs keratinocyte proliferation in vitro, no obvious abnormalities have been observed in epidermal-specific GLUT1 knockout mice under physiological conditions [52], indicating the existence of a compensatory mechanism in vivo. Whereas wound healing upon excisional injury and skin recovery following ultraviolet B (UVB)-induced damage is strikingly delayed in GLUT1-deficient mice [52], supporting the more important role of glucose uptake for cell proliferation in response to stress. Meanwhile, GLUT1 deficiency inhibits S6K phosphorylation [52], but the potential mechanisms remain to be further determined. Related to this, epidermal-specific AMPK deficiency leads to hyperactive mTOR signaling and increased cell proliferation after upon injury, UVB exposure, and phorbol ester application [54], suggesting the importance of mTOR-mediated energy metabolism in skin homeostasis.

mTORC1 activation is also regulated by amino acid availability, especially leucine and arginine [91]. However, how amino acid is sensed and signaled to mTORC1 in epidermis remains largely unclear. CD98hc (*Slc3a2*), a heterodimeric amino acid transporter positively modulates mTOR activity in vitro [92]. Boulter et al. demonstrated that *Slc3a2* deletion in mouse epidermis results in aberrant skin homeostasis, including delayed hair growth, wound healing and impaired keratinocyte proliferation [53], indicating a role of amino acid availability in skin homeostasis.

### 4.2. PI3K/AKT/mTOR Signaling Cascade in Epidermal Morphogenesis and Barrier Formation

Hyperactivated PI3K/AKT/mTOR molecular cascade has been extensively described in skin cancers and hyperplasia diseases [8,93]. Recent studies with conditional knockout mouse models highlight the importance of this pathway in epidermal morphogenesis and skin barrier formation.

PI3K-dependent kinase-1 (PDK1) is a serine/threonine, transmitting PI3K-dependent signaling to downstream kinases, such as AKT, in response to insulin/IGF-1 (Figure 2). During epidermal development, PIP3-PDK1 signaling occurs preferentially at the apical side of dividing basal cells. Deletion of PDK1 attenuates AKT activation and impairs basal cell asymmetric cell division, differentiation and stratification, resulting in perinatal lethality due to impaired barrier function [55]. Phosphorylating amino acid residue T308 of AKT by PDK1 is essential for its activation. Several studies identified that AKT is also critical for keratinocyte differentiation [94,95]. Accordingly, mice with AKT1 and AKT2 isoforms double knockout display impaired epidermal development and die soon after birth, possibly owing to respiratory failure [56]. However, mice with single AKT knockout survive and exhibit an apparently normal skin phenotype [57,58,60], suggesting that AKT isoforms might have compensatory functions in the skin. A more recent study reveals stratum corneum defects in AKT1 null mice due to less filaggrin [59]. In summary, the phenotypes of these mouse models suggest that PI3K-AKT signaling controls epidermal differentiation and barrier formation.

We and others recently demonstrated that mTOR complexes, as the core elements of the signaling cascade, are essential for embryonic epidermal development and skin barrier formation [61,62]. We generated mouse models with epidermal-specific disruption of mTOR, mTORC1 and mTORC2 by depleting *Mtor*, *Raptor* and *Rictor*, respectively [61]. Mice with epidermis-*Mtor* or *Raptor* ablation exhibit severe defects in epidermal morphogenesis and skin barrier formation during embryogenesis, resulting in neonatal lethality. Basal cell growth and proliferation are likely affected upon on mTORC1 inactivation [61,62]. Additionally, p18 is a lysosomal adaptor protein and is essential for mTORC1 activation in response to amino acids. The epidermal development and skin barrier formation are severely impaired in epidermis-specific p18 deletion mice [65]. Besides, loss of p18 in epidermis impairs filaggrin processing and leads to autophagosomes accumulation in epidermis, which resembles mTORC1 inhibition in many aspects [65].

In contrast, mice with epidermis-specific *Rictor* deletion can survive but exhibit epidermal hypoplasia and an abnormal differentiation process [61,64]. Mechanistically, inactivation of mTORC2 leads to impaired asymmetric cell division of the basal cells, which has also been observed in IGF1-R- and PDK1-deficient mice [55,61,80]. Our recent study shows that mTORC2-mediated AKT signaling in epidermis is critical for filaggrin processing and lipid metabolism [63]. Intriguingly, in AD patients, an increased RAPTOR level correlates with decreased AKT1 activity and impaired filaggrin processing, which is likely mediated by reduced protease cathepsin H (CTSH) [6]. All these studies emphasize the central role of mTOR complexes in skin barrier formation. Perturbed mTOR signaling in epidermis can drive skin diseases.

The activity of PI3K/AKT/mTOR signaling pathway can be induced by depleting their negative regulators genetically. PTEN inhibits mTOR activity by dephosphorylating PIP3 back to PIP2 [96] (Figure 2). In fact, PTEN mutations are implicated in Cowden disease, which is characterized by hyperkeratosis and papillomatosis in tissues, indicating a role for PTEN in skin homeostasis [97]. Mice with keratinocyte-specific PTEN deletion exhibit epidermal hyperplasia, increased hair follicle morphogenesis, tumorigenesis and enhanced re-epithelization during wound healing [66,67,68]. TSC functions as another negative regulator of mTORC1 through binding RHEB protein. TSC1 knockout mice exhibit embryonic lethality [98]. Squarize et al. developed a mouse model with conditional knockout TSC1 in epidermis, which displays increased epithelialization during wound healing [68]. Mice with epidermal-specific TSC1 deficiency exhibit skin hyperplasia, wavy hair and curly whiskers, and severe facial inflammation neonatally, resulting in an increased death rate [69].

### 4.3. mTOR Downstream Mediates in Epidermal Morphogenesis and Skin Barrier Function

Through their downstream effectors, activated mTOR signaling regulates a wide range of biological processes (Figure 2). In this section, we discuss the role of critical effectors and cellular processes downstream of mTORC1 and mTORC2 in epidermal morphogenesis and skin barrier formation.

#### 4.3.1. Protein Synthesis

mTORC1 promotes protein synthesis mainly by activating S6K and inhibiting 4E-BP [16]. Phenotypes of S6K1 or S6K2 mutants are considerably milder than those mutants of components of mTORC1 [70,71]. Mice deficient in S6K1 display a significantly reduced body size, whereas in mice with S6K2 loss, it tends to be less severe, and both are viable [70,71]. However, double knockouts of S6K1 and S6K2 display reduced viability owing to lethality before and after birth [72]. These studies suggest a feasible compensatory function between S6K1 and S6K2.

4E-BP-dependent translation regulates many physiological and pathological processes associated with metabolism. Two independent studies showed that 4E-BP1/2 double knockout mice are more sensitive to diet-induced obesity, indicating resistance to insulin when compared with wild-type mice [73,74]. Consistently, a more recent study provides evidence that triple knockout of 4E-BP1/2/3 mice are also prone to diet-induced obesity [75]. Besides, Shihyin Tsai group showed that skeletal muscle-specific overexpression of 4E-BP1 mice exhibited improved metabolic parameters and sensitivity to insulin [99]. Altogether, these studies demonstrate their critical roles of S6K and 4E-BP activities in regulation of cell proliferation and metabolism, however, their particular roles in skin epidermis remain to be determined in the future.

#### 4.3.2. Autophagy

Autophagy is characterized by formation of double-layer-membrane autophagosomes, which then translocate to lysosomes, where their cargo is degraded to prevent cell damage and provide energy for cells under conditions of starvation [100]. mTORC1 is the key regulator of autophagy via inhibition of autophagosome formation and autophagy genes by transcription factor EB (TFEB), and rapamycin treatment promotes this process [101]. Several lines of evidence suggest that autophagy plays a critical role in the integrity and homeostasis of epidermal cells [102,103]. mTORC1 inhibition induces epidermal autophagy, which indicates a possible role of mTORC1 in epidermal barrier defect diseases [102,104]. Intriguingly, ablating autophagy by deleting *Atg7*, encoding a key serine/threonine kinase required for autophagosome formation, has no obvious effects on epidermal differentiation and skin barrier function formation [76]. However, deleting *Atg7* likely suppresses carcinogen-induced protumorigenic inflammatory microenvironment and tumorigenesis of the epithelium [105]. Recently, Qiang and colleagues identified that mice with epidermis-specific deletion of *Atg7* have impaired wound closure [77]. However, our knowledge of the direct molecular link between mTOR and epidermal-autophagy in vivo is limited. However, these findings prompt us to suppose that mTOR-mediated autophagy may link to epidermis development and much more in vivo evidence would be needed.

#### 4.3.3. PKC

PKC, a family of protein kinase, can be phosphorylated by active mTORC2. Inactivation of mTORC2 results in reduced both PKC protein and phosphorylation levels [106]. To date, three PKC isoforms have been identified: cPKC (α, β, γ), nPKCs (δ, ε, θ, η) and aPKC (ζ, ι). With an in vitro human keratinocyte culture system, it has been shown that PKC isoforms have associations with keratinocyte differentiation programs [107]. PKCδ promotes keratinocyte differentiation by increasing Kruppel-like factor 4 protein expression, which drives involucrin gene transcription [108]. PKCα expression is restricted to the granular layer and inhibiting its expression leads to suppressed loricrin and filaggrin expression [109]. Experiments with mouse models have also illustrated roles of PKC in cutaneous inflammation. Mice with epidermal-specific overexpression of PKCα display a seemingly normal epidermis, however, those mice are more sensitive to the treatment of 12-O-tetradecanoylphorbol-13-acetate (TPA), a classic mouse cutaneous tumor promoter, which results in intraepidermal inflammation and disruption of the epidermis [78]. Overexpression of another PKC isoform, PKC-epsilon (PKCε), can similarly enhance sensitivity to both chemical stimuli and UVR, promoting the development of squamous cell carcinoma [79].

#### 4.3.4. Transcriptional Regulation

Transcription factor forkhead-box protein O1 (FoxO1) is regulated by IGF-1 via AKT-dependent phosphorylation, thereby resulting in nuclear exclusion and proteasomal degradation of FoxO1 [110]. p63 gene is known as a transcription factor controlling epidermal development, stratification and differentiation [111,112]. Attenuated IGF-1R/AKT signaling leads to FoxO1 retaining in the nucleus, where it binds and inhibits p63 transcriptional activity. Consistently, mice with transgenic expression of a constitutive nuclear FoxO1 variant in the epidermis show a fragile and translucent skin phenotype and exhibit neonatal lethality due to the impaired epidermal stratification [80].

Activated mTORC1 can promote the translation of transcriptional factors such as Hypoxia-inducible factor-1 alpha (HIF-1α). HIF-1α is a ubiquitously distributed transcription factor involved in multi cellular and physiological processes [113]. It is well recognized that activation of mTOR potently enhances the activity of HIF-1α, which promotes angiogenesis by stimulating VEGF-A secretion [114]. HIF-1α is also important in skin physiology and pathophysiology [115]. In steady state, epidermis is mildly hypoxic and expresses substantial HIF-1α. Interestingly, epidermal deletion of HIF-1α retards systemic hypoxic responses by decreasing the expression of renal erythropoietin (EPO), an erythropoietic agent, in oxygen-depleted conditions, thus indicating the critical role of skin in systemic physiological response [81]. The significance of HIF-1α has also been demonstrated with in vitro cultured human keratinocytes. HIF-1α overexpression leads to enhanced keratinocyte proliferation and migration [116,117]. As a consequence of impaired proliferation and migration of keratinocytes, epidermal-specific HIF-1α knockout in a mouse model disturbs wound healing [82]. In contrast, HIF-2α deficiency accelerates wound repair [83], thus supporting the notion that these isoforms probably have distinct physiological effects and reflecting the non-redundancy between HIF [118,119].

MYC is a key transcription factor positively regulated by mTOR. Recent studies underline its importance in skin homeostasis and several mouse models have been generated to explore its effects on epidermis [120]. In mice, epidermis-specific MYC constitutive expression leads to hyperplasia of epidermis, delayed wound closure and gradual development of spontaneous tumors [84,85]. Later, Zanet and colleagues developed a mouse model with MYC deletion from epidermis. In this case, MYC deletion leads to impaired wound healing and premature differentiation of epidermal cells, consequently thinning the epidermis and resulting in a tight and fragile skin [86]. However, one must notice that the role of MYC in epidermis is complex, very much depending on the cell type used and cell layer in which MYC is expressed in the context [120].

#### 4.3.5. Lipid Metabolism

The epidermis is an active site of lipid metabolism. During keratinocyte differentiation, epidermal lipids are synthesized and extruded into extracellular space to form a lipid matrix, composing a protective epidermal barrier together with cornified keratinocytes in the cornified layer. Disturbed lipid metabolism and altered lipid composition often lead to skin barrier defects [121]. Emerging evidence suggests that mTOR complexes play an important role in regulating lipid biogenesis, including epithelial cells [122,123]. mTORC1 regulates lipid metabolism mainly through two transcription factors: PPAR and SREBP. PPARα- and PPARβ-deficient mice show cutaneous repair defects [87]. However, the role of mTORC1 signaling in epidermal lipid metabolism remains largely unclear. Interestingly, PPARα activation can normalize the epidermal lipid ratio and promotes the skin barrier of both normal skin and filaggrin-deficient skin [124,125,126]. Therefore, PPAR activators may be potentially applied in treating AD patients by promoting keratinocyte differentiation and lipid homeostasis. mTORC1 also positively regulates SREBP, which belongs to the family of basic helix-loop-helix leucine zipper transcription factors and regulates the expression of enzymes involved in lipid and cholesterol metabolisms [35]. Interestingly, studies in human keratinocytes showed that SREBP-1c mRNA expression is induced during human keratinocyte differentiation [127]. SREBP-2 is a predominant form expressed in keratinocytes. However, its role in the transcriptional regulation of the lipid synthetic for epidermal barrier formation and restoration is not yet clear.

Activated AKT triggers expression of a number of lipogenic genes [128]. Interestingly, we have recently shown that epidermis mTORC2-deficient mice exhibit reduced epidermal lipid synthesis, resulting in a thinning, translucent, and fragile skin [63]. Therefore, it is tempting to speculate that the mTORC2-AKT axis might play a critical role in epidermal lipid metabolism, but the underlying mechanisms need to be further defined [63].

## 5. Concluding Remarks and Perspectives

Epidermal barrier function is established and maintained through a complex and highly coordinated stratification program. Studies over recent years have identified mTOR signaling networks, including IGF-1/IGF-1R, PI3K/AKT and mTOR complexes as critical mediators in epidermal barrier formation and homeostasis. These signaling cascades regulate protein and lipid synthesis, transcription, and cytoskeleton reorganization, which in turn impact keratinocyte proliferation, cell polarity, and the stratification program. Their genetic inhibition abrogates and impairs epidermal morphogenesis and skin barrier formation. Increased mTOR activity, on the other hand, may result in skin cancer and other hyperplasia skin diseases. As a result, it appears that a delicate balance of mTOR signaling activity is critical for skin health and their dysfunction or dysregulation might be implicated in the pathogenesis of a variety of skin diseases.

In recent years, a wealth of knowledge has accumulated on the understanding of the nutrition signaling network in regulating cell growth and metabolism [129]. New regulators of mTOR signaling pathway, such as amino acid sensors, have been recently identified [130]. Unraveling the activities of these upstream and downstream mediators in the skin environment will be intriguing and should improve our understanding of the complex regulation of epidermal barrier function, which will eventually allow for selective manipulation of mTOR signaling activity and function. In addition, recent research sheds light on how other signaling pathways including Hippo, WNT and Notch, influence mTOR [131]. Many of these pathways are essential for the programs of epidermal stratification, patterning and differentiation during skin morphogenesis. As a result, it is crucial to elucidate the molecular mechanisms connecting mTOR and the other regulators during epidermal development and homeostasis. Furthermore, recent studies have disclosed an unexpected, reciprocal interplay between mechanics and the crucial metabolic signaling, including mTOR [132]. The epidermis is an excellent example of a polarized tissue. The polarity machinery, cellular adhesion and mechanical forces are coordinated to shape epidermal development and morphogenesis [133]. As mentioned, IGF-1/PI3K/mTORC2 signaling axis is crucial for epidermal stratification and barrier formation. Therefore, an interesting goal for the future will be the identification of the cross-talk between mechanics and mTOR signaling during epidermal morphogenesis, as it may help to better understand the molecular and cellular mechanisms underlying the epidermal barrier formation and the development of skin disorders.

Lastly, beyond epidermal keratinocytes, it is equally important to determine the specific roles of the mTOR signaling network in the other skin cell types, including but not limited to immune cells, fibroblasts or adipocytes. Given the hyperactivation of mTOR signaling in many pathological conditions, such as cancer and psoriasis, their roles in pathogenesis of skin diseases are now well recognized. A better understanding of the complex network of molecular players in skin pathologies will ultimately be important for exploring strategies for medical therapy.

## Figures and Tables

**Table 1:** Skin phenotypes of mouse models with modified mTOR signaling and its up-downstream mediators.

Gene	Genetic Modification	Epidermal Barrier	Additional Phenotypes	Refs
**Ligands and receptors**				
*Igf1*	Whole body knockout	n.d	Epidermal hypoplasia/neonatal lethality	[47]
	Epidermal-specific overexpression	n.d	Epidermal hyperplasia/spontaneous tumor formation in aged mice	[48]
*Igf2*	Whole body knockout	n.d	No obvious abnormalities	[47]
*Igf1/Igf2*	Whole body knockout	n.d	Epidermal hypoplasia	[47]
*Ir*	Whole body knockout	+	No obvious skin phenotype	[49]
	Epidermal-specific knockout	+	No obvious abnormalities/decreased epidermal thickness	[50]
*Igf-1r*	Whole body knockout	+++	Translucent skin/thin epidermis/neonatal	[47]
	Epidermal-specific knockout	++	Epidermal hypoplasia/impaired skin barrier	[51]
*Ir/Igf-1r*	Epidermal-specific knockout	+++	Epidermal hypoplasia/neonatal lethality	[50]
*Glut1*	Epidermal-specific knockout	+	No obvious skin phenotype/delayed wound healing	[52]
*Slc3a2*	Epidermal-specific knockout		Hair growth delay/impaired skin wound healing	[53]
**Kinase and scaffold proteins**				
*AMPK*	Epidermal-Specific knockout	+	Epidermal hyperplasia upon injury and UVB exposure	[54]
*Pdk1*	Epidermal-Specific knockout	+++	Epidermal hypoplasia/impaired skin barrier function/neonatal lethality	[55]
*Akt*	AKT1/AKT2 whole body double-knockout	n.d	Defects in skin development/translucent skin/neonatal lethality	[56]
	AKT1 whole body knockout	+	No obvious abnormalities/stratum corneum defects	[57,58,59]
	AKT2 whole body knockout	+	No obvious abnormalities	[60]
*Mtor*	Epidermal-specific knockout	+++	Epidermal hypoplasia/impaired skin barrier function/neonatal lethality	[61]
*Raptor*	Epidermal-specific knockout	+++	Epidermal hypoplasia/impaired skin barrier function/neonatal lethality	[61,62]
*Rictor*	Epidermal-Specific knockout	++	Epidermal hypoplasia/impaired skin barrier function	[61,63,64]
*P18*	Epidermal-specific knockout	+++	Impaired skin barrier function /neonatal lethality	[65]
*Pten*	Keratinocyte-specific knockout	n.d	Epidermal hyperplasia/tumor formation/enhanced re-epithelization during wound healing	[66,67,68]
*Tsc1*	Epidermal-specific knockout	n.d	Increased re-epithelization during wound healing	[68]
			Epidermal hyperplasia/wavy hair and curly whiskers/hair lose	[69]
*Rheb*	Epidermal-specific knockout	+++	Epidermal hypoplasia/impaired skin barrier function	[62]
**Downstream effectors**				
*S6k*	S6K1 whole body knockout	n.d	Small body size/increased life span	[70,71]
	S6K2 whole body knockout	n.d	No obvious phenotypic abnormalities	[70]
	S6K1/S6K2 whole body double knockout	n.d	Reduced viability/neonatal death	[72]
4E-BP	4E-BP1/2 whole body double-knockout	n.d.	Increased sensitivity to diet-induced obesity	[73,74]
	4E-BP1/2/3 whole body triple-knockout	n.d	Increased sensitivity to diet-induced obesity	[75]
*Atg7*	Epidermal-specific knockout	+	No obvious skin phenotype/impaired skin wound healing	[76,77]
*Pkc*	PKCa overexpression in epidermis	+	No obvious phenotypic abnormalities/increased sensitivity to TPA	[78]
	PKC-epsilon over expression in epidermis	+	Mild abnormalities/ more sensitive to TPA	[79]
*FoxO1*	Overexpression of nuclear variant in epidermis	+++	Epidermal hypoplasia/impaired stratification/neonatal lethality	[80]
*Hif*	HIF-1a epidermal-specific knockout	n.d.	Epidermal aging/pruritic inflammation/delayed wound closure	[81,82]
	HIF-2a epidermal-specific knockout	n.d.	Accelerated wound closure	[83]
*Myc*	Overexpression in epidermis	n.d.	Epidermal hyperplasia/spontaneous tumor/delayed wound closure	[84,85]
	Epidermal-specific knockout	+++	Epidermal hypoplasia /tight and fragile skin/impaired wound healing	[86]
*PPAR*	PPARα whole body knockout	n.d.	delayed wound healing	[87]
	Heterozygous PPARβ mutant	n.d.	delayed wound healing	[87]

Skin barrier defect is depicted as severe (+++), mild (++) and normal (+). n.d. = not determined.

## Data Availability

Not applicable.
